# Meaningful Moments of Connection: How People Affected by Dementia and Their Carers Living at Home Understand, Interpret and Experience Everyday Aesthetics

**DOI:** 10.1002/gps.70136

**Published:** 2025-08-03

**Authors:** Sarah Fox, James Thompson, John Keady

**Affiliations:** ^1^ Division of Nursing, Midwifery and Social Work The University of Manchester Manchester UK; ^2^ NIHR Applied Research Collaboration Greater Manchester Manchester UK; ^3^ Global Brain Health Institute Trinity College Dublin Dublin Ireland; ^4^ School of Arts Languages and Cultures The University of Manchester Manchester UK; ^5^ Greater Manchester Mental Health NHS Foundation Trust Manchester UK

**Keywords:** care, connections, dementia, everyday aesthetics, identity, moments

## Abstract

**Objectives:**

This study considers how people affected by dementia living in their own homes understand and interpret everyday aesthetics and what relevance this holds in their everyday lives.

**Methods:**

Nine households, comprising ten family carers and seven people living with dementia, shared their self‐identified meaningful moments of connection reflective of their personal understanding and interpretation of everyday aesthetics. Data collection involved a range of creative and self‐initiated approaches, including scrapbooking, photography, and elicitation interviews. Data were analysed using reflective thematic analysis.

**Results:**

Six discrete but interlinked themes were identified, namely: (1) Connection with others; (2) Connection with materiality; (3) Connection with self‐image; (4) Connection with pride and societal value; (5) Connection with enjoyable activities; and (6) Connection with the lived environment, that revealed how people affected by dementia self‐identify everyday aesthetic experiences in their daily lives.

**Discussion:**

Our findings show these self‐identified experiences span multiple physical and psychological domains of everyday life, each of which acts to support the personhood and identity of the person living with dementia. We suggest that development of a new model of care based on everyday aesthetic needs and informed by people affected by dementia, might bridge the gap between theory and practice in person‐centred care. Going forward, developing care practices and support systems which focus on identifying and fulfilling the aesthetic needs of people living with dementia may offer a novel way to enhancing independence and personal well‐being.

## Key Points

- The language of everyday aesthetics is unfamiliar to the public and can be misinterpreted. However, the theoretical concept can be understood by a lay audience if accessible language is used. Working together with a study advisory group we suggest that ‘meaningful moments of connection’ may be used as an accessible proxy term when discussing everyday aesthetics with a lay audience.
- People living with dementia and their carers can identify meaningful moments of connection within their everyday lives. These experiences span participant's sensory, physical, relational and cognitive environments and are based on their biographical histories, experiences and preferences.
- Meaningful moments of connection identified by those living with dementia or carers are linked with identity for the person living with dementia. Therefore, we suggest that personhood and a sense of identity can be supported when a person living with dementia is able to engage with such moments.
- Everyday meaningful moments of connection are created, maintained, and protected by the actions of the person living with dementia, the actions of those who care for them and the resources available to them.
- The development of a new model of care based on meaningful moments of connection and informed by people living with dementia might bridge the gap between theory and practice in person‐centred care. Specifically, understanding which experiences are meaningful for a person living with dementia and developing a new model of care focused on everyday actions which can create and maintain situations which bolster identity, personhood and wellbeing through access to these meaningful moments of connection.

## 1 Introduction

There are approximately 55 million people living with dementia worldwide with around one million individuals in the United Kingdom (UK) alone, this is predicted to increase to over 1.6 million by 2050 [1]. Symptoms range from memory deficits to sensory processing difficulties, problems with planning, communication and activities of daily living. These symptoms impact the physical and emotional health of those affected, changing the way they engage with, find meaning in, and view their self and their identity in their everyday lives [1, 2, 3].

There are currently no accessible disease modifying therapies or standardised post‐diagnostic support pathways across the UK, meaning that most people with dementia continue to live at home relying upon an inconsistent offering of community support initiatives and family care [4, 5]. It is therefore unsurprising that seven of the top ten research priorities identified by carers and people living with dementia via the James Lind Alliance focus on care [6]. Indeed, the top research priority named in the report focused on maintaining the person living with dementia's independence for as long as possible in all care settings, which included their own home.

Since the late 1980s to the present day and grounded largely in the seminal work of Professor Tom Kitwood, person‐centred care practices have been a ‘gold standard’ for psychosocial dementia care, focusing on maintaining independence for people living with dementia and supporting individual personhood [7]. Indeed, in the UK, the most recent NICE dementia guidelines [8] include reference to best practice in person‐centred care and support for people living with dementia, their families, and carers. However, the extent to which Kitwood's psychosocial theories in dementia have been reliably translated into global practice is still debated. For example, a study by the Health Policy Partnership [9] observed that person‐centred care lacked conceptual clarity and a common definition, which hindered implementation. Interestingly, Tieu [10] recently expanded upon this uncertainty to suggest that person‐centred care has become ‘a buzzword which is open to multiple interpretations and definitions, rendering it devoid of meaning’ (p.2). Such observations suggest that the concept needs a unifying model to ensure its values are transferable, reproduceable, and applicable in the context of everyday care environments.

In response to this dilemma, we look to the field of everyday aesthetics and the crucial role it plays in shaping our everyday lives. While aesthetics is often associated with art and beauty and discussed in isolation from everyday life, Saito [11] suggests that everyday aesthetics offers a deeper understanding of how we actively engage with and respond to our environments, defining the concept as the ‘aesthetics of objects, environments and situations from our everyday life’ [12]. The psychological impact of everyday aesthetic experiences will vary from person to person dependent on their personal history and preferences, and it has been suggested that these experiences can impact an individual's subjective wellbeing [13]. For example, an individual who holds fond memories of a childhood spent on a farm may find comfort in the smell of livestock, while an individual with no biographical connection to that environment might experience the same smell as unpleasant.

Fleetwood‐Smith et al. [14] argued that better appreciating everyday aesthetic and sensory experiences may be particularly important for people living with dementia. Indeed, because dementia impacts several cognitive domains, including communication, planning and perception, it often leaves an individual's ability to experience everyday aesthetics through sensory knowledge intact [15], thereby increasing the importance and utility of everyday aesthetics for those living with dementia. Indeed, this can be seen in a range of studies which have investigated and found value in aesthetic experiences and approaches in dementia care settings including care homes and specialist mental health assessment wards for people living with dementia [14, 16, 17, 18, 19, 20, 21].

Given the potential for everyday aesthetic experiences to impact subjective wellbeing, their theorized importance for people living with dementia and the observation that the emotional tone created by everyday aesthetic experiences is unique to the individual, we suggest that everyday aesthetic experiences represent an under investigated aspect of person‐centred care. Specifically, understanding how people living with dementia experience their moment‐to‐moment lived environment through the theoretical framework of everyday aesthetics might open new ways of thinking about person‐centred practice, connecting with and enhancing independence and wellbeing in a range of care settings. This study therefore aims to consider how people affected by dementia living in their own homes understand and interpret everyday aesthetics in their lives and what meaning this holds for them.

## 2 Methods

### 2.1 Study Design and Data Collection

#### 2.1.1 Public Involvement

Our intention to explore everyday aesthetics in the lives of people affected by dementia was frustrated by confusion surrounding the term ‘aesthetic’ since the term is not commonly used in the public sphere. This concern was highlighted in the first meeting of our study advisory group. This group consisted of one white British female living with dementia who attended all meetings, one white British male carer who attended the first and third meeting and a black British female carer who attended the first meeting only. Our first meeting focused on the theory of everyday aesthetics and aims of this study, while the next two meetings covered study methodology, participant‐facing materials and recruitment. All three study advisory group meetings took place at the start of the project before data collection began.

Study advisory group members were unaware of the meaning of ‘everyday aesthetics’, with one member questioning whether the term was associated with the cosmetic or beauty industry. To reframe everyday aesthetics in a language that was understandable to the study advisory group, all authors and advisory group members discussed the meaning of everyday aesthetics, including accessible examples based on Saito's previously shared definition of everyday aesthetics [12]. Through our discussions, our study advisory group recommended when explaining this study to our participants, we replace the term ‘everyday aesthetics’ with ‘meaningful moments of connection’. The study advisory group felt that the term ‘meaningful’ was an acceptable proxy for aesthetic, since it captured a sense of heightened sensory and emotional awareness while not necessitating the experience to be explicitly novel or beautiful. Moreover, the use of ‘moment’ reflected that these heightened sensory experiences are transitory. Finally, the term ‘connection’ indicated that the moments were based on a point of connection between individuals, or between an individual and their lived environment, including objects, sensations, and physical and cognitive experiences.

Together with the authorship, members of the study advisory group met a further two times during the study design phase and helped to design all participant‐facing materials used in the study.

#### 2.1.2 Data Collection

Data was collected through a combination of creative social research methods which included multisensory scrapbooking and elicited interviews focused on scrapbook entries [22]. Scrapbooking offered the person living with dementia and their family carer an opportunity to document their meaningful moments of connection as, or soon after, they occurred, avoiding explicit need for participants to recall experiences retrospectively. Furthermore, we focused on methodological flexibility and inclusive study design which facilitated the engagement of people living with dementia either living alone or with family carer(s) (where that was the existing relationship), study participant's data were ordered and analysed as households.

In our study, physical scrapbooks consisted of (i) semi‐structured pre‐printed pages including prompt questions; (ii) a polaroid camera to capture images of moments; (iii) an audio recorder to capture sounds associated with a moment; and (iv) a pocket in the scrapbook to store related artifacts. Scrapbook pages also included prompts to encourage participants to reflect on the feelings attached to a moment, the experience(s) which catalysed it, a narrative of the experience, and whether the moment was shared with anyone else (see Supporting Information S1 and S2). Participants could therefore choose to document their self‐identified meaningful moments of connection in traditional written format, through polaroid photographs, using a voice recorder, sketching, by collecting related artifacts, or via a mixture of these formats.

Participants were invited to take part in up to three interviews to discuss their scrapbooked meaningful moments of connection. Interviews were planned around participants' schedules and personal commitments; these took place on average once a fortnight. In the week(s) preceding the interview, participants used their scrapbooks to document their meaningful moments of connection, and the number of moments collected between interviews ranged across participants. Interview length ranged from one to two hours, guided by participant engagement and the number of moments collected in their scrapbooks. Interviews were undertaken by the first author and took place in participants' homes. When scrapbook entries were present, interviews focused on open ended questions and reflections elicited by entries. When no scrapbook data was available, interviews focused on ascertaining what experiences in the preceding weeks participants retrospectively identified as being meaningful.

### 2.2 Participants, Recruitment and Ethics

The study was conducted in the North‐west of England and all interviews were conducted in participant's homes between autumn 2023 and spring 2024. Participants were recruited to the study through the first author's face‐to‐face presentations at community groups, with support from several community leaders and charity organisations. Community groups were accessed based on familiarity with research team and advisory group members. To increase sample diversity, groups tailored specifically to local Black and Ethnic Minority populations (BAME) and LGBTQ + communities were also targeted. Overall, seven community groups were accessed. Table 1 outlines the study participants, including participant demographics and characteristics, all names are pseudonyms in line with the ethical approval protocol.

**TABLE 1:** Participant demographics.

Household #	Number of interviews	Participant pseudonym	Gender	Age	Ethnicity (self identified)	Relationship	Mode of study engagement
1	2	Simon	M	90	White british	Person living with dementia	Scrapbook/Interview Photos Written entries
	3^a^	Rose	F	60	White british	Carer (wife)	
2	3	Jack	M	74	White british	Carer (husband)	Scrapbook/Interview Photos Written entries Audio clip
		Flora	F	78	White british	Person living with dementia	
3	3	Clive	M	71	Jewish	Person living with dementia	Interview only
		Ruth	F	76	Jewish	Carer (wife)	
4	3	Frank	M	78	White british	Person living with dementia	Scrapbook/Interview Photos Written entries
		Liz	F	78	White british	Carer (wife)	
5	2	Ray	M	85	Black british	Person living with dementia	Scrapbook/Interview Photos Written entries
		Leena	F	74	Black british	Carer (wife)	
		Tom	M	56	Black british	Carer (son)	
		Michael	M	60	Black british	Carer (son)	
6	2	Nelly	F	66	Black british caribbean	Carer (daughter)	Scrapbook/Interview Photos Written entries
7	2	Aisha	F	41	Muslim	Carer (daughter)	Interview only
8	2	Garth	M	64	White british	Person living with dementia	Scrapbook/Interview Photos Written entries
		Lilly	F	64	White british	Carer (wife)	
9	2	Sky	Non‐binary	40	Black british	Person living with dementia	Scrapbook/Interview Photos Written entries

*Note:* Table showing participant pseudonyms, familial relationships, demographics, age and mode of study engagement.

^a^ Simon was taken to hospital during the study period so one interview was with Rose alone.

Ethical approval was obtained from the Health Research Authority Social Care Research Ethics Committee (IRAS reference: 326052). In line with the requirements of the Mental Capacity Act 2005 [23] the person living with dementia's capacity was assessed prior to study consent during an initial home visit (see Supporting Information S3 for the capacity assessment used in this study). Following our ethical approval protocol, all participants were required to have the capacity to be included in the study. However, as capacity can fluctuate in people living with dementia, we used the process consent model [24] during longitudinal engagement to ensure that the person living with dementia remained aware of the research aim and ambition of the study. To do this, at the start of each home visit, the first author re‐established consent by talking through the study aims, expected outcomes and individual rights as study participants.

### 2.3 Data Analysis

Audio recordings of interviews were manually transcribed; transcripts were combined with written entries and photographs, where present, for further analysis. Transcripts and scrapbook entries were reviewed in full several times by SF and continually discussed with co‐authors JT and JK to reflect on the content and analytical categorisations. Reflexive thematic analysis was used as the guiding framework [25] informed by the first author's reflexivity (see statement at the end of the article).

Following the analytical process identified by Braun and Clarke [25] we identified 40 codes ranging from meaningful moments of connection to food, play, nature and social connections. These codes were further categorised into six themes which reflected the emotional, physical, sensory and embodied aspects of the moments collected by our participants. Each theme is expressed as a type of connection to an aesthetic aspect of everyday life, from how participants connect with material objects to their connection with the lived environment. The six identified themes were: (1) Connection with others (including codes such as, family and pets); (2) Connection with materiality (including codes such as clothing and sentimental objects); (3) Connection with self‐image (including codes such as humour and culture); (4) Connection with pride and societal value (including codes such as talents and work); (5) Connection with enjoyable activities (including codes such as singing and gardening); and (6) Connection with the lived environment (including codes such as towns and nature).

Each theme will now be addressed in turn using a range of methods to further explore their meaning and value. The theme numbering is not intended to be hierarchical.

## 3 Results

### 3.1 Theme 1: Connection With Others

The data showed that, for people living with dementia, meaningful moments of connection often focused on their relationships with others which included family, friends, and pets. Connections with others held value because they generated feelings of safety, comfort and a way to maintain connection with their own identity. For example, Sky (household 9), who lived with young onset dementia, described spending time with their parents as an experience which grounded them and reminded them of their own identity:

I feel like I’m fighting in a jungle and you lose yourself. Sometimes there’s no one to ground you, remind you of who you are and where you come from and yeah, they [your parents] don’t tell you they … just … just being around them makes you know, you know.

However, social connections were not always positive. This was the case for Aisha (household 7), a family carer who expressed frustration in social situations when her mother would ask people ‘*who they are and how she knows them’*. She did not want anyone who she and her mother were not *‘very close with’* to know about her mother's diagnosis, which appeared to impact her mother's ability to engage in social relationships outside the family home. However, when Aisha's mother was able to connect with trusted family friends in the safety of their home, she experienced positive emotions which Aisha described as happiness and through the onomatopoeic sound ‘*Ahhh*’ which, in this exchange, referred to a feeling of contentment and relaxation. This example shows the importance of safe connections with trusted peers for a person living with dementia, but also highlights difficulty when balancing care for a person living with dementia with a carer's wish not to disclose a diagnosis:

SF Does she like chatting to your friend's mom?

Aisha Yeah. At those times she will be very, very happy, ‘Ahhh’ like that. Someone is coming, it's good

SF After the visit, does your mum stay a little bit happier?

Aisha Yeah. Yeah. She will be very happy for a few days

Positive connections were not limited to friends and families, indeed several participants highlighted meaningful moments of connection which centred around their pets. Rose (household 1) described a moment when the family cat ‘*decided he wanted to join his dad in bed*’, exclaiming that she ‘*didn't think the cat could have got any closer’.* As Rose described this moment Simon smiled at being referred to as a ‘*cat dad’*, suggesting happiness in this mutual affection and his identity as the cat's ‘dad’.

This theme touches on how relational ties can help people living with dementia to preserve a sense of identity and happiness. Such moments of connection may also offer an island of reprieve amid the everyday challenges a dementia diagnosis brings, where individuals may, in Skys words, ‘*feel as though they are fighting, and losing themselves’*.

### 3.2 Theme 2: Connection With Materiality

This second theme reflects meaningful moments of connection which relate to material objects or items, this includes relation to the physical and sensory properties of these objects. Objects discussed by participants in the study ranged from sentimental trinkets, books, photographs, food and clothes. These objects often reflected an individual's history, connecting them with people, places, preferences or previous versions of themselves. Sky (household 9) reflected on the importance of being able to walk around their home in a pair of heels, and how these shoes helped them feel connected to their non‐binary identity. Their father also had a dementia diagnosis and, in relation to both their father and their diagnosis, they discussed the overall importance of collecting cherished mementos ‘*while you still can’*. These collections proactively connected people living with dementia to their present and historic identity, as this extract from the data reveals:

I think when people get diagnosed with dementia, it should be in whatever manual that now’s the time to ask your family member parts of their persona, parts of their identity, who they were, so you can start gathering things that are sentimental to them that’s important to them that are key tokens. […] this is the box that’s gonna have all your mementos, in the future things that you like, that you know, that you always cherish, that if I show you in the future you’ll come back to me. It’s like a light in the dark, you know show me the heels and I’ll be like ‘Oh, yes, that’s it’. But if you don’t plan for my memory loss, then I’ll just be lost. Sky (household 9)

**FIGURE 1:**
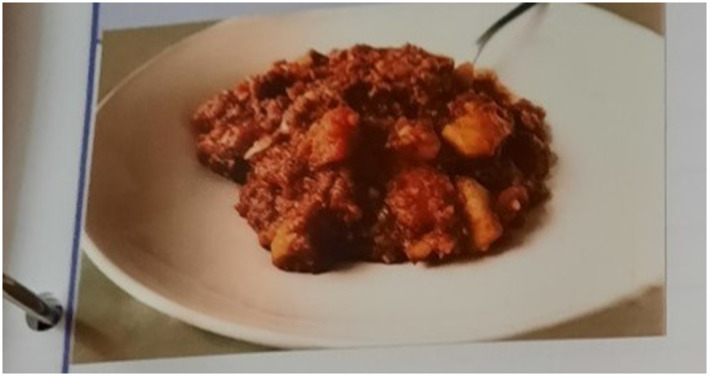
Flora's Corned Beef Hash. This image was captured by Jack on the polaroid camera included in the study material and was affixed into his scrapbook next to text describing the meal and its meaning for the couple.

Meaningful moments of connection were also derived from the sensory aspects of materials, for example through smells and tastes. For carers like Jack (household 2), concerns over his wife Flora's lack of appetite meant that many of their moments centred around food, particularly the types of food that Flora enjoyed eating (see Figure 1).

Drawing on Figure 1, Jack observed in his scrapbook that certain meals were meaningful for himself and Flora, as this scrapbook entry illustrates:

Flora’s favourite meal corned beef hash, we made a pan full which lasted for two days meals. Flora loves stews but corned beef hash is definitely number one these days. I have to encourage Flora to eat but she loves when I suggest making it and always clears her plate.

Interestingly, Jack also experienced a meaningful moment of connection when preparing this meal for Flora. The dish held autobiographical meaning and resonance for the couple as Jack told SF that he introduced Flora to this meal when they were first married.

### 3.3 Theme 3: Connection With Self‐Image

Self‐image can fluctuate following a dementia diagnosis as changes in the brain impact upon an individual's capacities, social presentation and the way others respond to them. In this study, several participants found meaning in moments which allowed them to re‐connect with their pre‐diagnosis self‐image. These were moments which reflected how they viewed themselves and how they wanted to be seen by others. For example, Clive (household 3) discussed feeling angry and frustrated in the moment he received his dementia diagnosis and how these feelings of anger sat at odds with the way he identified as being a ‘*nice person’*:

*So, er she* [referring to the memory clinic neuropsychologist] *asked me: ‘What’s that, what’s that, what’s that?’ I knew exactly what they were. But I couldn’t bring it out. I thought, ‘That’s crazy’. Went to the doctor. He said, ‘Sit down. Don’t do this, don’t do that, don’t do the other’ and I was f‐ing and blinding. I was getting really*. *uptight when I was doing it* [referring to his interaction with the doctor]*. I thought, ‘What the hell am I doing? I’ve got to get this right. I’m a nice person’.*

In line with his self‐image of being a ‘*nice person’*, many of Clive's meaningful moments of connection were linked to kind and helpful things he did within his neighbourhood, suggesting that being nice and helpful brought him personal meaning and value.

A further illustration of this phenomenon was expressed by Nelly (household 6). Nelly's mother, who lives with dementia, raised two children and worked as an auxiliary nurse in a maternity unit. From Nelly's descriptions of her mother, it came across that caring was an important part of her mother's career and identity prior to her diagnosis. During the conversation, Nelly observed that her mother still connects with those around her through acts of care. Her mother ‘*does a lot of caring for her adult children’* asking them if they have taken their medication and enquiring a lot about their health and wellbeing. However, Nelly's mother's attempts to care for her adult children are not always well received, particularly by Nelly's brother. Nelly bought her mother a doll as an outlet for her to reconnect with her self‐image and identity as a carer. The act of caring for the doll helped Nelly's mother to calm down when she's ‘*a bit frazzled’*. Moreover, the doll also acted as a point of connection between Nelly and her mother, opening dialogue and bringing humour to their relationship.

### 3.4 Theme 4: Connection With Pride and Societal Value

Many participants reflected on moments when their skills and knowledge were valued by others and how this value gave them a sense of pride and purpose; potentially restoring some of the confidence they had in their societal value, which their diagnosis brought into question. Garth (household 8) observed how receiving a diagnosis made him feel discarded, but that meaningful engagement as a lay advisor on research projects restored his identity as someone with a purpose and role in society:

Well, it’s, it makes you feel wanted, you know, it’s wanted, valued. Because obviously I had to take early retirement from work when I was given me diagnosis and you feel like you’ve been discarded, for want of a better phrase, and because of the research work that I’m involved in, I do feel all the things that, you know, I used to feel negative about, you know, it used to make me feel very depressed and things like that.

However, unfortunately, most people living with dementia struggle to find work or activities which bring them pride and a sense of societal purpose [26]. For those who don't have the opportunity to engage in structured ‘work’ following a diagnosis, many found pride in daily accomplishments or in reflecting on previous accomplishments. Simon, Clive and Ray (households 1, 3 and 5 respectively) expressed pride in winning at games and activities, with Simon and Ray both discussing their skills at dominoes while Clive proudly recalled a story about correcting a question on a quiz, suggesting that next time ‘*if they* [the community group organisers] *wanted some help* [planning a quiz]*, they should come to him’*.

Many participants also reminisced fondly about their previous careers, and how valued their skills were when they were part of the workforce. Reminiscence is an ‘in the moment’ activity, and the act of reminiscing on a past accomplishment or achievement, which brought with them a sense of pride, appeared to reactivate those positive feelings in the present moment.

### 3.5 Theme 5: Connection With Enjoyable Activities

This theme relates to activities and hobbies participants highlighted as being meaningful moments of connection in their everyday lives. Participants shared a range of activities that they enjoyed, most being linked to their autobiographical histories and preferences. Participants shared activities, from trips to familiar restaurants and cafés, to holidays, gardening, watching movies and baking.

The lives of those affected by dementia can be limited by both their condition and the resources available to them, meaning that people living with dementia sometimes become cut off from the meaningful experiences which scaffold and support their unique identities. In this study we observed examples of individuals who were no longer able to access such activities; alongside examples where carers were able to support an individual's unique preferences. For example, Frank and Liz (household 4) used to attend a local social club together, but Liz observed that Frank is no longer willing to come with her. While Frank spoke fondly about the club, saying that they do ‘*decent dinners*’ and that it’s ‘*pretty good really’*, when asked why he won't come anymore he simply stated that he ‘*just doesn't fancy it’*. As the conversation continued Frank revealed insecurities regarding the club, specifically a concern that he ‘*won't be able to keep up with the conversations'*, which is ‘*a situation he doesn't like’*. This contrasts with the dementia support group they both attend, which Frank enjoys, saying that the members of that club are ‘*all in the circumstances of the same’*.

Tom, who cares for his father Ray (household 5), provided another example when he spoke about how Ray tended to become distressed while at the family home, but that attending a local community dementia group has improved his mood:

*He’d sit and cry, rocking and crying, then if you ask him why he won’t tell you […]. But when he’s been going out to the centre* [a local community group for African Caribbean people living with dementia] *he enjoys it. And sometimes he might not remember that he’s been but there’s a definite improvement in how he feels. There’s some spillover from that and I think that’s where that emotional bit comes in, maybe you know there’s some sort of echo there, you know a feeling of… a memory maybe*.

Tom suggests here that the in the moment enjoyment Ray experiences at the group ‘*echoes and spills over*’ into his time outside the group, which in turn improves his overall mood.

In another interview, Simon (household 1) revealed his love of fishing as a hobby. This activity held significance for him and, despite verbalising little during our time together, he came to life when speaking about fishing; performing the actions associated with casting a line and verbalising the sound of the lure moving through the water ‘*pshpshpsh’*:

SF I was going to ask, Simon, what is it you like about fishing?

Simon I don't know, it's just fishing, I enjoy it so much […]

SF So what's your technique? I've never fished before, how do you do it?

Simon *It depends what you're fishing for, trying to catch roach? It's with a pole* [does casting actions with hands]*. You have to find the depth of the water, just fish a bit, three inches off the bottom that's the best way and ground bait attract them and just get it on and carry…pshpshpsh. I've had some great fishing times*

Engaging with this beloved hobby enabled Simon to experience a continuity of his identity, as this was an activity he learnt about as a young boy. The sensory and embodied way he described the act of fishing during the interview suggests that reflecting on the physicality of this activity was an immersive ‘in the moment’ experience for him, which brought him joy. Simon's wife Rose supported him to engage in aspects of this activity even when his mobility became a limiting factor. She supported him to organise and clean his fishing tackle, added fishing‐related mementoes around their living space to trigger memories and conversation and, when appropriate, supported him to fish at a small private lake.

Supporting access to meaningful activities, as Rose, Tom and the care group did for Simon and Ray, may be important to enable people living with dementia to maintain a connection with their preferences and identities.

### 3.6 Theme 6: Connection With the Lived Environment

Many participants discussed moments which reflected how connections with their lived environments held meaning for them. These experiences ranged from connection to the natural world to time spent in urban spaces or at home. Several participants discussed how their connection to certain places is linked to their biographical history and identity, while others noted that they valued accessible safe places they could enjoy despite mobility problems and cognitive impairments.

**FIGURE 2:**
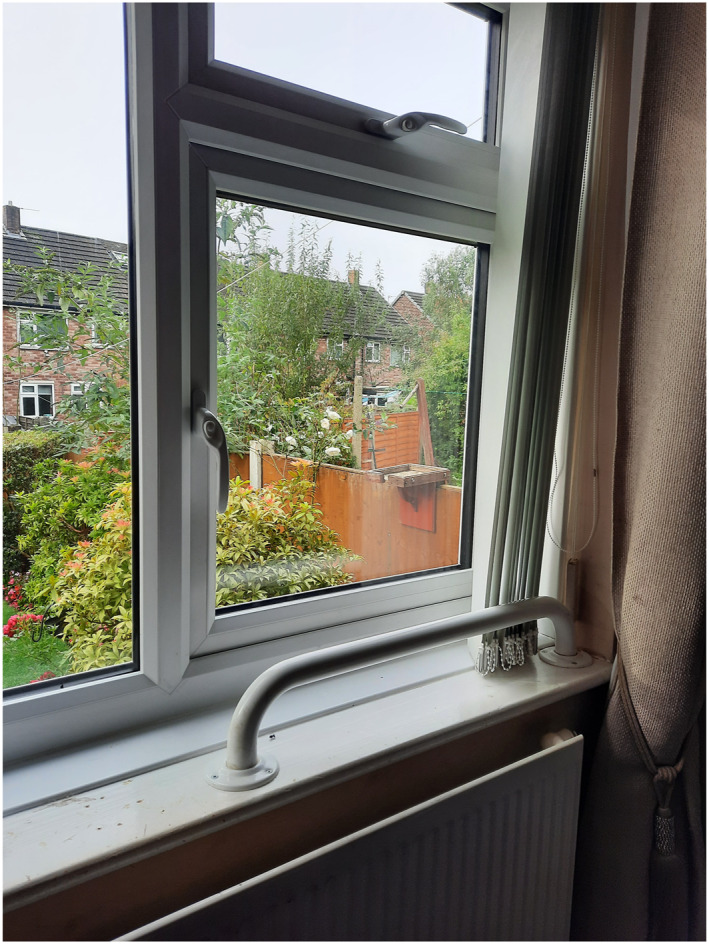
Grab bars for garden viewing. This image was captured by researcher SF, following a conversation with Rose regarding her installation of grab bars at the bedroom window which enabled Simon to watch the garden birds.

When Simon's (household 1) deteriorating health meant that he was unable to leave the home, his wife Rose ensured that their bedroom space was modified with grab bars which enabled Simon to raise himself up from the bed and watch birds feeding at a table outside their bedroom window, an activity they often enjoyed together. This adaptation is shown in Figure 2.

Alongside connections with home and nature, communities and retail environments were also often discussed by our participants. Frank (household 4) talked about how he found meaning in being able to fix things and mentioned several recent do‐it‐yourself (DIY) tasks he had undertaken around the home. Alongside his interest in DIY, Frank also reflected on how much he enjoyed being able to travel to and browse local DIY stores. Frank valued being able to access this type of retail space and, in this conversational extract, he reflects on how shopping trips took his mind off things:

SF Well, it's funny how those things become a bit of a day out, don't they?

Frank Well, it does

Liz Yeah

SF On rainy days

Frank And it takes your mind off things. And you enjoy doing what you're doing

Liz Yeah

Frank Your mind goes somewhere else dun't it. You enjoy it

Frank's statement suggests that access to everyday retail spaces takes his mind off some of the challenges he faces, offering an immersive ‘in the moment’ experience which allows his mind to go somewhere else and links to his personal identity as a handyman.

While each theme offers a unique reflection on the type of everyday aesthetic experience underpinning a meaningful moment of connection, we also observed that several moments drew from more than one theme. For example, Frank's interest in DIY (an enjoyable activity) drew him to enjoy visiting hardware stores (part of his lived environment). This is reflective of how aesthetic needs are multifaceted, built from an individual's lived experience, preferences, and history, and therefore do not exist in isolation. This thematic intersection does not undermine the unique elements of our themes, that is the ability of a hardware store to take Frank's ‘mind off things’, is distinctive to that aspect of his lived environment while also drawing from his biographical connection to DIY.

## 4 Discussion

This paper describes how people affected by dementia experience meaningful moments of connection in their everyday lives and how these might be reflective of everyday aesthetic experiences. The data captures six categories of meaningful moments, each centred upon a different type of connection; 1. Connection with others; 2. Connection with materiality; 3. Connection with self‐image; 4. Connection with pride and societal value; 5. Connection with enjoyable activities; 6. Connection with the lived environment. The self‐identified meaningful moments of connection highlighted by our participants emerge from a range of, predominantly intersubjective, physical and psychological experiences. The types of everyday experience which trigger these moments vary between participants, reflecting their personal history, culture, connections and preferences. For example, Flora's love of corned beef hash is linked to a connection with her husband Jack, this being a meal he introduced her to, while the meaning Clive derives from helping others draws from his wish to be seen as a nice person. Therefore, a common theme running across our data is that each meaningful moment, through a type of everyday connection with people, places, memories and objects, acts to support the individual living with dementia's unique identity; identity being linked with the individual's history, culture, connections and preferences.

While we recognise that debate is ongoing regarding the definition of what constitutes everyday aesthetics [11, 27, 28], we would like to draw attention to two points which we believe make our dataset relevant to Saito's [11] theoretical stance on everyday aesthetics. The first draws on this study's novel focus on supporting people living with dementia and their care partners to self‐define moments in their life which they interpret as being meaningful. Saito [11] suggests that in contrast to traditional art, everyday aesthetic non‐art objects and experiences are ‘frameless’ making the observer the creator of the aesthetic object, defining for themselves what aspects of their everyday lives are aesthetic. Thus, assuming our study advisory group's simplified definition of everyday aesthetics as meaningful moments of connection is theoretically aligned with Saito's [11, 12] stance on everyday aesthetics, supporting our participants to self‐identify their own meaningful moments of connection also fulfils the notion that the ‘observer is the creator of the aesthetic object’. In other words, the study participants selected from a range of everyday experiences those which they self‐defined as being meaningful (or aesthetic) to them.

Secondly, our dataset contains several instances where meaning is drawn predominantly from mental rather than bodily experiences, for example through reminiscence. Such experiences do not align with some definitions of everyday aesthetics as being sensory embodied experiences. This raises the question whether mental rather than physically embodied meaningful moments, such as reminiscence, can be considered aesthetic. To address this we suggest that, while reminiscence does rely on memory, it also holds sensory and embodied elements. Specifically, in our study, moments of reminiscence involved active listening and engagement from another person, in this case a researcher. This interaction reflects an ‘in the moment’ sensory experience of connection to another person. Also, as we see when Simon reminisced about fishing, a memory can reactivate the physical sensory elements of a prior experience. Simon exemplified this through the act of mimicking the physical action of casting a line and through the utterance he made to reflect the sound of a lure moving through the water. Thus, we argue that these primarily cognitive meaningful moments of reminiscence can also be thought of as sensory, embodied, aesthetic experiences.

Drawing on theories of personhood, which often underpin person‐centred care practices, we can link our findings to the work of Mittner who, drawing on Barad [29, 30], suggests that an individual's personhood and identity is built and continually reconfigured based on an interplay between external connections and internal processes. Like Mittner and Barad's theory, Hughes' [31] situated‐embodied‐agent view of personhood also suggests that:

We are situated in multifarious dimensions of culture, personal history, social context, as well as moral and spiritual fields. Part of our embodiment means that we engage with these dimensions of our world through our senses, as well as by our understanding.

(p. 1412)

Both theories highlight how connection to an embodied sensory, spiritual, cultural and cognitive world (an aesthetic world), are important for an individual's identity, and subsequent personhood. Therefore, based on our data we suggest that personhood and a sense of identity can emerge from everyday aesthetic experiences (in our study ‘meaningful moments of connection’) which are unique to an individual's history, social context, cultural background and so on. Our six themes may therefore be viewed as part of a web of everyday aesthetic moments which share a common role in drawing on physical and psychological connections to bolster identity and personhood.

Our data also provides insights into how such moments come about in the day‐to‐day lives of people affected by dementia. We see that these meaningful moments of connection are created, maintained and protected by the actions of the person living with dementia, the actions of those who care for them and the resources available to them, at home and in their local communities. For example, when Garth (household 8) had to leave his job, he lost his sense of societal value, but through his choice and the availability of opportunities to engage in dementia research he found a new community who valued his incite. Simon's (household 1) connection to his love of fishing was maintained and protected by his wife Rose, who curated moments through which Simon could re‐connect to this part of his identity. Caring connections also extend beyond family and friends into the community. Indeed, access to a local community group, tailored to his African Caribbean heritage, enabled Ray (household 5) to experience connection to his heritage and to connect with others who share this identity.

Our findings suggest that meaningful moments of connection can offer a platform for people living with dementia to maintain a connection to their own personhood and identity. These moments do not need to be overtly artistic or creative but must hold aesthetic value for the person who experiences them. These findings argue that, while therapeutic art interventions undoubtedly play an important role in health and wellbeing for those living with dementia, significant personal benefit may also be gained from acknowledging and supporting people living with dementia to engage in their own everyday aesthetic worlds. As verbal and cognitive capacities change, leading to limited physical capacity for people living with dementia, we may still be able to support their access to meaningful moments of connection, such as a certain food, social interactions, activities or item of clothing. This may continue to provide connection and stabilise personhood and identity as the disease progresses [14, 29]. Indeed, documenting such preferences early in the diagnostic process may provide a practical repository of meaningful moments of connection which may be further drawn upon at later stages of the condition to support person‐centred care and link into existing care models such as the VIPS framework and dementia care mapping [32].

### 4.1 Study Limitations

While our sample covered a diverse population, including several participants from seldom heard communities, our participants were not geographically diverse, coming only from predominantly urban regions of the North‐west of England. We also acknowledge that not all aesthetic experiences are positive. In this study we focused on positive experiences, however everyday aesthetic experiences can be both positive and negative, and both can impact on an individual's wellbeing [13]. Going forward, understanding the circumstances surrounding and impacts of negative experiences is equally important for people living with dementia and their carers.

## 5 Conclusion

This study shows that people living with dementia experience and understand aesthetics in their everyday lives through meaningful moments of connection, and that these experiences have a positive impact on their sense of identity and personhood. Several studies already explore a range of aesthetic practices in dementia care, either explicitly (e.g., sensory and embodied practices in care settings [33, 34] and via materiality of care [16]), or without direct reference to aesthetics (e.g., music for dementia [35] or lived environments tailored to enriching everyday experiences [36]). To the best of our knowledge, a model of care has yet to be proposed which focuses on identification of a broad spectrum of everyday aesthetic needs and preferences valued by people living with dementia and their carers, initiated soon after diagnosis. We suggest that policy makers in the UK acknowledge the variety of aesthetic experiences which are important for people living with dementia, and endeavour to increase accessibility to a broader range of spaces, places, services and experiences. This may be achieved through workforce training and provisions which open a wider range of activities, spaces and services up to people living with dementia who may otherwise struggle to access these due to both physical and cognitive impairments.

## 6 Reflexivity Statement

The first author's academic background is in quantitative methods, specifically in neuroscience and electrophysiology. Prior to undertaking this project, she worked for the National Health Service focusing on public engagement and involvement in the context of policy development with and for people living with dementia. As a public involvement specialist, SF viewed participants as experts through their own experience, this informed the process of working with an advisory group to develop a lay definition of everyday aesthetics. During data collection, SF encouraged participants to self‐identify and reflect on their own meaningful moments of connection, rather than guiding conversations toward a prescriptive type of experience. Finally, SF's background in dementia policy steered data analysis and conclusions to coalesce around concepts with implementable outcomes and policy implications.

## 7 Future Research

We would like to explore the breadth of aesthetic moments and their impact on participants identifying across a range of sexualities, geographies, cultural and gender identities. We would also like to expand this research to explore the impact of negative aesthetic experiences. Ultimately, we believe this work will lead to the development of a novel care model which could be initiated soon after diagnosis and support the person living with dementia throughout their dementia journey across all care settings.

## Conflicts of Interest

The authors declare no conflicts of interest.

## Supporting information

Supporting Information S1

Supporting Information S2

Supporting Information S3

## Data Availability

The data that support the findings of this study are available on request from the corresponding author. The data are not publicly available due to privacy or ethical restrictions.
