# Supplementary material for: Meaningful Moments of Connection: How People Affected by Dementia and Their Carers Living at Home Understand, Interpret and Experience Everyday Aesthetics
Source: Int J Geriatr Psychiatry. 2025 Aug 3;40(8):e70136. doi: 10.1002/gps.70136 (PMC12319174; doi:10.1002/gps.70136)
Supplement: Supplementary file 1 — Supporting Information S1 [file GPS-40-e70136-s001.pdf]

# Meaningful moments of connection: A study exploring the impact of meaningful everyday experiences for people living with dementia and their care partners.

MANCHESTER 1824
The University of Manchester

NIHR | Applied Research Collaboration Greater Manchester

**What is this study about?:** Our lives are filled with meaningful moments, from the smell of a home cooked meal to the purr of a cat as it lays on your lap. These day-to-day experiences may not be life-changing, but they can still lift our spirits and brighten our days. These moments are also important for people living with dementia and their care partners, helping them connect with the things that are important for them and offering a positive anchor to help them move through challenging times.

Therefore, we have designed this study to investigate the ways that people living with dementia and their care partners experience and create meaningful moments in their everyday lives and why these moments are important to them.

**How are we studying this?:** Participants, people living with dementia and care partners, will be asked to keep a scrapbook of meaningful moments they create or encounter in their day-to-day lives. Participants will be interviewed about the moments they have collected.

**Why is it important?:** Research suggests that keeping track of meaningful experiences and moments in our daily lives and revisiting these may help us to feel more positive about our life-circumstances and ourselves.

We hope that being part of this study will help participants to recognise and reflect on positive experiences and that this may help them to navigate through more challenging times. We also want to understand what day-to-day activities and experiences are important for people living with dementia and their care partners. This knowledge will help us to work with health and social care providers to improve well-being support and provisions for people living with or caring for someone living with dementia.

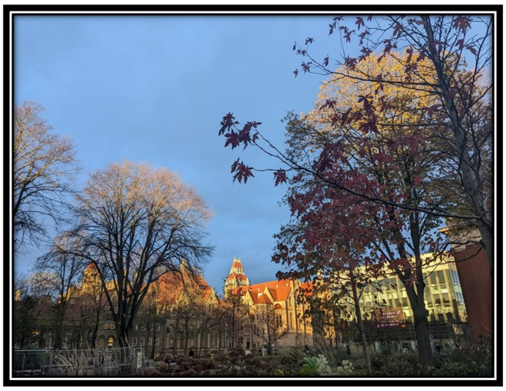

Alzheimer's Society United Against Dementia

# Meaningful moments of connection: A study exploring the impact of meaningful everyday experiences for people living with dementia and their care partners.

**Meaningful moments:**

A meaningful moment can be anything which feels important or meaningful to you, to get you started we've provided a few examples below—these examples have come from our research team and public involvement team (including current carers and people living with dementia).

**Here are a few examples:**

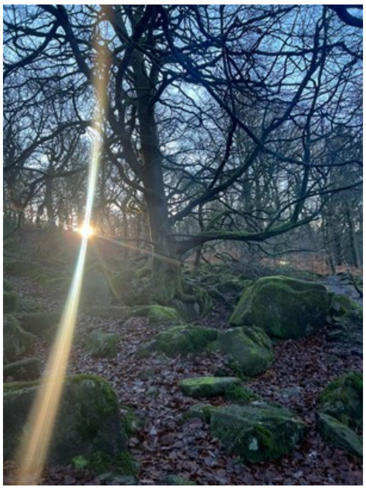

- I took this picture during a walk on new year's day close to where we live. Being here in this moment helped me feel a connection to nature and the beauty of the natural world. And the transformative power of the sun.

- My dad was an excellent gardener, but these days he can't do as much. One day, while I was tending to the garden, doing the heavy stuff, he came outside with me, laid on the patio and started to pick all the tiny weeds which were growing up through the cracks. He was so happy to be out working in his garden and absorbed in the task, I could see that it brought him so much joy.

- Since my knee replacement I've started running again. I ran from my house to a group I attend, over 5km away. It ended up being quicker than waiting for the bus, even though I got a bit lost on the way. It felt like such an achievement for me, it was a joy.

# Meaningful moments of connection: A study exploring the impact of meaningful everyday experiences for people living with dementia and their care partners.

## How to use the camera:

### Taking pictures

Your camera will come pre-loaded with **10 sheets** of photographic film, so you will instantly be able to start taking pictures. We will provide you with extra film in case you run out.

**Follow the instructions on the next page to load new film into your camera if you run out**

---

**Camera Front**

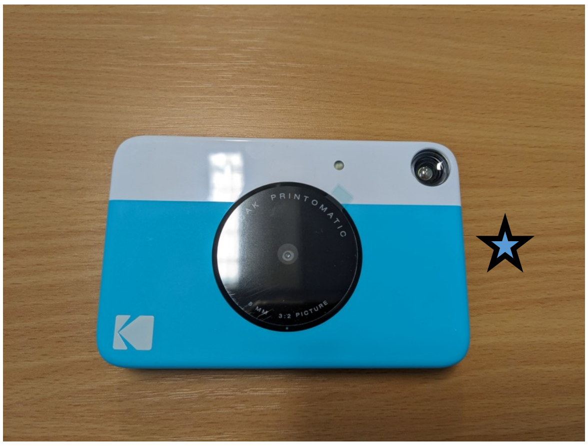

**Camera Back**

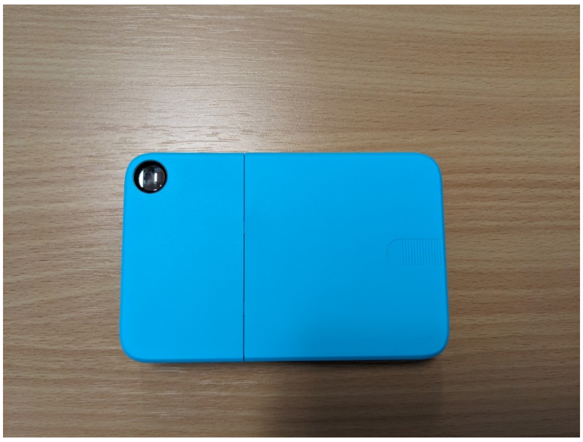

**Camera Top**

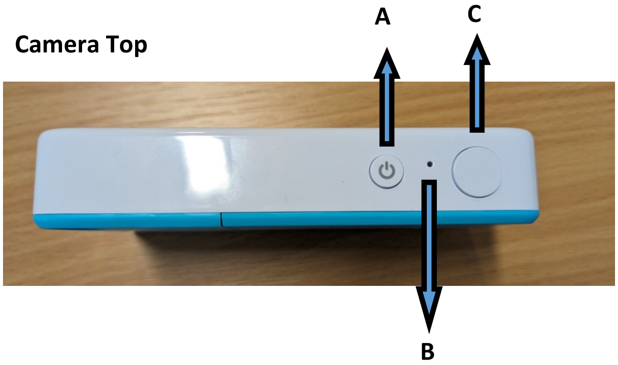


**Camera Bottom**

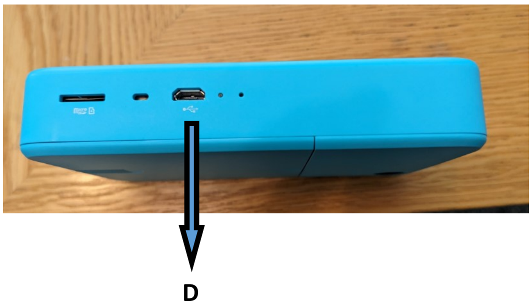


---

**Turn on and off:** Press and hold **button A** to turn your camera on and off, when the camera is on **light B** will glow white.

**Charging:** If your camera does not turn on when you press and hold **button A**, it may need to be charged. Connect your charging cable to **slot D** and plug the camera into a plug socket. Charging can take up to 1 hour.

**Taking a picture:** Look through the viewfinder on the back of the camera, centre what you want to take a picture of in the middle of your view and press **button C**. The camera will make a beeping noise, wait for a few seconds and the picture will start to develop. The picture will come out of the hole on the side of the camera (**location indicated above by the blue star on first picture)**

## How to use the camera:

### Loading film

Your camera will come pre-loaded with 10 sheets of photographic film, so you will instantly be able to start taking pictures. We will provide you with extra film in case you run out.

**Follow the instructions below to load new film into your camera if you run out**

Picture 1:

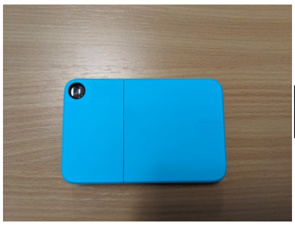

Picture 2:

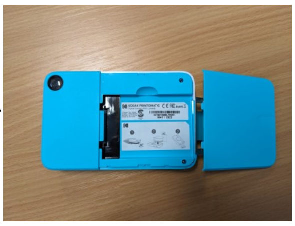

Turn the camera round so its back side is facing up (Picture 1)

Side off the side panel to reveal the tray for loading photographic paper (Picture 2)

Picture 3:

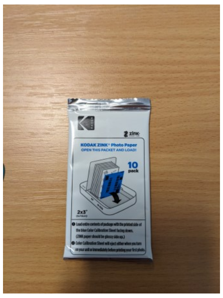

Open a new pack of 10 sheets of photographic paper. Try not to touch the centre of the paper (hold at the side as in picture 4)

Picture 4:

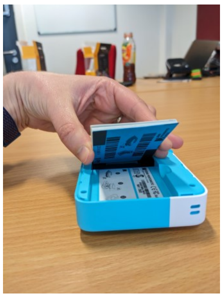

Picture 5:

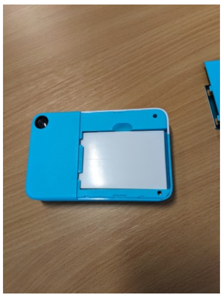

Load the photographic paper into the paper tray, make sure to load with the cardboard sheet (with the bar code) facing down (pictures 4/5)

Picture 6:

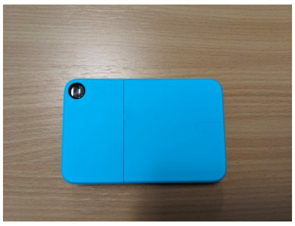

Slide the back panel back into place

After loading the photographic paper into the camera and closing the back panel, turn the camera on and take one picture, this will eject the cardboard sheet (with the barcode).

**You are now ready to start taking pictures with your camera**

# Meaningful moments of connection: A study exploring the impact of meaningful everyday experiences for people living with dementia and their care partners.

## How to use the Multi Memo voice recorder:

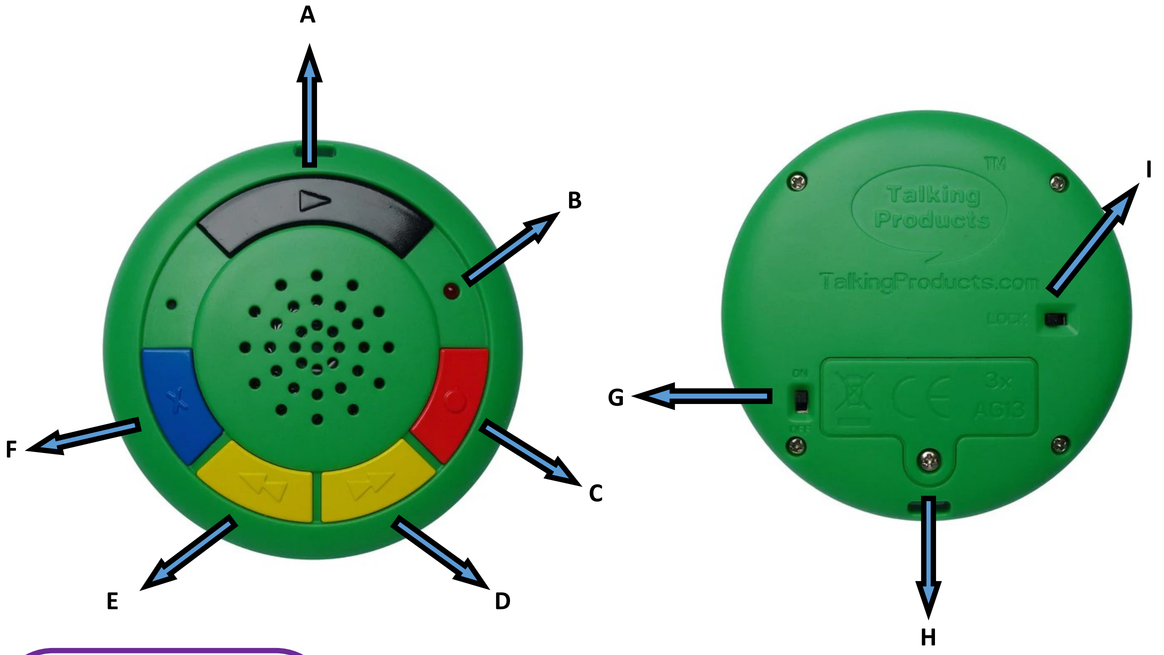


**A:** Play button

**B:** Record light

**C:** Record button

**D:** Next Button

**E:** Previous Button

**F:** Erase Button

**G:** ON/OFF switch

**H:** Battery compartment

**I:** Lock switch

**On/Off:** To turn device on or off, toggle the ON/OFF switch **(G)** on the back of the device.

**Record:** Press and hold the record Button **(C)** to start recoding, release to stop recording. The recording light (B) will glow red while you are recording. Total recording time is 6 minutes and you can record up to 60 individual messages.

**Play back:** Press the play button **(A)** once to play back your last recorded message. Press the next **(D)** and/or previous **(E)** buttons to skip between messages. To skip to the latest message press and hold the next button **(D)** for 3 seconds, to skip to the first message press and hold the previous button **(E)** for 3 seconds.

**Erase a message:** Locate the message you wish to erase using the next **(D)** and previous **(E)** buttons, then press the erase button **(F)** once—the recode light will flash and the device will beep to confirm the message has been deleted.

Lock: There is a lock switch **(I)** on the back of the device. If you slide this switch to the left position the erase **(F)** and record **(C)** buttons will both be disabled.
