# Supplementary material for: Meaningful Moments of Connection: How People Affected by Dementia and Their Carers Living at Home Understand, Interpret and Experience Everyday Aesthetics
Source: Int J Geriatr Psychiatry. 2025 Aug 3;40(8):e70136. doi: 10.1002/gps.70136 (PMC12319174; doi:10.1002/gps.70136)
Supplement: Supplementary file 2 — Supporting Information S2 [file GPS-40-e70136-s002.pdf]

My Moments

Date: ________________________

Photos: please stick photo(s) here

Please describe what happened:

MANCHESTER
1824
The University of Manchester

My Moments

Date: ______________________

Was anyone with you at the time?

How did it make you feel?:

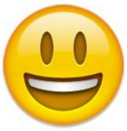

Happy

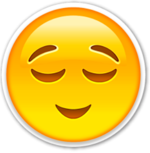

Contented

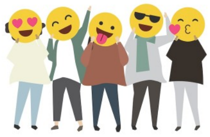

Connected

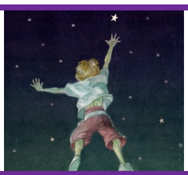

Inspired

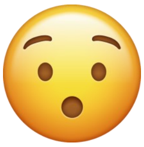

Surprised

Or something else, please describe your how this made you feel here:

MANCHESTER 1824
The University of Manchester

My Moments

Date: ______________________

Extra notes, sketches or reflections

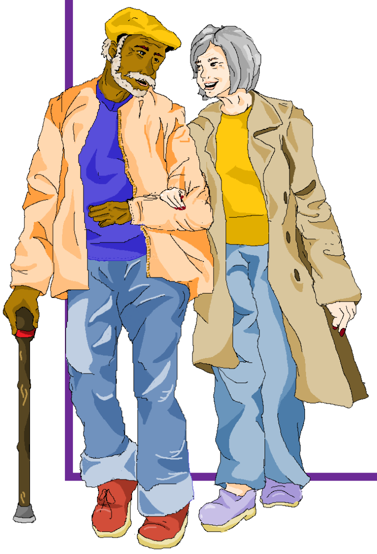

MANCHESTER 1824

The University of Manchester
