# Supplementary material for: Meaningful Moments of Connection: How People Affected by Dementia and Their Carers Living at Home Understand, Interpret and Experience Everyday Aesthetics
Source: Int J Geriatr Psychiatry. 2025 Aug 3;40(8):e70136. doi: 10.1002/gps.70136 (PMC12319174; doi:10.1002/gps.70136)
Supplement: Supplementary file 3 — Supporting Information S3 [file GPS-40-e70136-s003.docx]

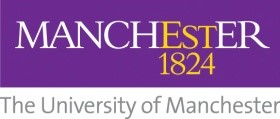


**Moments of connection: A study exploring the impact of meaningful everyday experiences for people living with dementia and their care partners.**

**Mental Capacity Assessment Form**

Notes:

1. In line with the Mental Capacity Act (2005) and the DEEP Dementia Voices ethical gold standards for dementia research, we aim to include (not exclude) people living with dementia in our study and assume that people have capacity to be involved (unless it is shown that they don’t).
2. In advance of completing this form we will work closely with participants living with dementia and their care partners (if partners are involved) to understand their cognitive requirements. We will use this information to ensure that materials we present are tailored to their needs i.e:
   1. Visual prompts
   2. Easy-read materials
   3. Verbal descriptions
   4. Sufficient time to consider the project and ask questions.
3. Although this form will be used at the time of recruitment to assess capacity, we also accept that consent is an ongoing and flexible process and that capacity of those living with dementia can fluctuate. Therefore, we will provide routine reminders and recaps for participants to reconsider and reflect on their involvement at all meetings. Participants will be prompted to reconfirm their consent and reassured that they are free to withdraw at any time.

| Participant name:  Researcher name:  Family member / care partner name(s) (if they are also involved in the assessment):  Date of assessment: |  | |
| --- | --- | --- |
| Describe the decision that the participant is making: | The decision is …………………….. | |
| Step 1: Get to know the person with dementia and the usual ways they would communicate consent to other activities in their everyday life and document this. | Record salient points …………………… | |
| Step 2: Involve family member(s) / carer(s), or other persons if present and as appropriate, to further explore how the person with dementia would usually communicate consent (or non-consent) in their everyday life. | Record salient points ………….. | |
| Step 3: Provide further support to help the person with dementia make the decision / maximise capacity. | Record salient points ………….. | |
| Step 4: Assessment of capacity  Q1 Is the person able to understand information relating to his / her involvement in the study?  Q2 Is the person able to sufficiently retain information related to his / her involvement in the study?  Q3 Is the person able to use or weigh-up the information in relation to making the decision?  Q4 Is the person able to communicate their decision by whatever means? | Yes / No  Give evidence or examples:  Yes / No  Give evidence or examples:  Yes / No  Give evidence or examples:  Yes / No  Give evidence or examples: | |
| Step 5: Decision taken  Given the responses to Questions 1 to 4, do you consider that the individual has the capacity to make an informed choice?  [Note: If you have answered ‘No’ to any of the questions in Step 4, then the person does not have the capacity to make this specific decision at this time]. | *I consider that the person has the capacity to make the decision.*  *I consider that the person does not have the capacity to make the decision.*  *I consider that it is appropriate to delay this assessment until the person is better able to demonstrate their capacity.* |  |
